# Key drivers of biomedical innovation in cancer drug discovery

**DOI:** 10.15252/emmm.201404596

**Published:** 2014-11-24

**Authors:** Margit A Huber, Norbert Kraut

**Affiliations:** 1 Department of Dermatology and Allergic Diseases, Ulm UniversityUlm, Germany; 2 Oncology Research, Boehringer Ingelheim RCV GmbH & Co KGVienna, Austria

## Abstract

Discovery and translational research has led to the identification of a series of “cancer drivers”—genes that, when mutated or otherwise misregulated, can drive malignancy. An increasing number of drugs that directly target such drivers have demonstrated activity in clinical trials and are shaping a new landscape for molecularly targeted cancer therapies. Such therapies rely on molecular and genetic diagnostic tests to detect the presence of a biomarker that predicts response. Here, we highlight some of the key discoveries bringing precision oncology to cancer patients. Large-scale “omics” approaches as well as modern, hypothesis-driven science in both academic and industry settings have significantly contributed to the field. Based on these insights, we discuss current challenges and how to foster future biomedical innovation in cancer drug discovery and development.

## Definition and translation of new therapeutic concepts

As exemplars of biomedical discoveries that have transformed the cancer treatment landscape, we will discuss recent therapeutic advances in malignant melanoma, non-small-cell lung cancer (NSCLC), acute myeloid leukemia (AML), and certain gynecological cancers.

In 2002, activating mutations in the gene encoding the serine/threonine kinase BRAF were identified by the Cancer Genome Project (Sanger Centre, UK) in a subset of human cancers, including about 50% of malignant melanomas (Davies *et al*, 2002). Less than 10 years later, the small-molecule kinase inhibitors vemurafenib and dabrafenib were approved for use in *BRAF* mutation-positive melanomas, as the front-runners in a competition between numerous companies engaged in cancer drug discovery (Bollag *et al*, 2012; Hauschild *et al*, 2012). As these compounds were made available to academic investigators (see Box 1), a broad range of studies emerged that investigated the mechanism of BRAF inhibition in *BRAF*-mutant tumors. Indeed, new insights, including on the activation of downstream pathway components such as MEK, have informed a recently approved targeted BRAF/MEK inhibitor combination of dabrafenib/trametinib (Flaherty *et al*, 2012).

**Box 1: Sharing of lead compounds with academia**

Providing open access to “probe compounds” with attractive features such as potency, selectivity, cellular permeability, and ideally also suitable physicochemical properties to enable *in vivo* studies is an increasingly attractive concept for the pharmaceutical industry. Such probes are valued reagents in both fundamental and applied biological research and are key tools in drug discovery that allow preclinical target validation in both academic and industrial laboratories. Early sharing of lead compounds with academia may help the industry to prioritize between different therapeutic concepts, explore new disease links including patient selection strategies, inform effective combinatorial approaches, and gain insights into potential resistance mechanisms—highly valuable information that would fuel further translational research. Often, companies will not immediately publish the clinical candidate, but rather describe a closely related compound with very comparable features. Thus, sensitive structural data remain undisclosed, helping to mitigate perceived risks toward protection of intellectual property. For example, in the case of BRAF inhibitors, important new biological insight into the mechanisms of resistance and MAPK pathway activation was initially revealed with the BRAF inhibitor PLX4720 (Tsai *et al*, 2008), a compound closely related to PLX4032 (RG7204, vemurafenib) which was only later made available to the scientific community for preclinical studies, and subsequently became the first marketed BRAF inhibitor (Bollag *et al*, 2012).

In 2004, two Boston-based academic groups discovered EGFR mutations in NSCLC, one through a systematic kinome-directed sequencing approach (Paez *et al*, 2004), and the second thanks to a hypothesis-driven *EGFR* sequencing effort (Lynch *et al*, 2004). Both groups were able to link the presence of *EGFR* mutations with an outstanding sensitivity of mutation carriers to EGFR tyrosine kinase inhibitors (TKIs). This provided the basis for successful biomarker-guided clinical development of the two selective EGFR TKIs gefitinib and erlotinib, as well as the irreversible ErbB family blocker afatinib (Tartarone *et al*, 2014). About 60% of EGFR TKI-treated patients become resistant by developing the EGFR T790M mutation, and based on early clinical trials appear to respond well to next-generation mutant-selective/wild-type-sparing EGFR inhibitors such as AZD9291 and CO-1686 (Jänne *et al*, 2014; Sequist *et al*, 2014).

Using a functional genomics approach, a Japanese team in 2007 discovered a chromosomal translocation resulting in an aberrant fusion gene that encodes a constitutively active ALK kinase in a subset of NSCLCs (Soda *et al*, 2007). A mere 3 years later, dramatic clinical responses were reported in ALK-translocated NSCLC patients upon treatment with crizotinib (Kwak *et al*, 2010), followed by its FDA approval in 2011. Even though this drug was developed initially as a MET kinase inhibitor and only later recognized to potently block ALK activity, these remarkable timelines to successful clinical translation and approval were only made possible through close academic/industry collaborations. As is the case with other targeted therapies for oncogene-addicted tumors, resistance to crizotinib is inevitable and is observed in less than a year (Camidge *et al*, 2012). Importantly, ceritinib, a second-generation ALK TKI approved by the FDA earlier this year, appears to be effective against many of the known resistance mechanisms that arise in patients exposed to crizotinib (Shaw *et al*, 2014). Over the past several years, multiple molecular mechanisms of resistance to targeted therapies have been discovered, resulting in the emergence of several common themes (see Box 2).

**Box 2: Predicting drug resistance to cancer drugs**

Resistance to targeted therapies is commonly categorized as either primary (i.e. intrinsic) or secondary (i.e. acquired). Primary resistance indicates a direct lack of a treatment response, while acquired resistance is defined by disease progression following an initial response. Acquired resistance may be mediated by target modifications (e.g. secondary mutations or gene amplification of the target that will abrogate the inhibitory activity of the drug), “bypass signaling” resulting in reactivation of parallel and/or downstream signaling pathways, or histologic transformation. For EGFR TKIs, all of the above mechanisms of acquired resistance have been described clinically, including a secondary threonine-to-methionine substitution within the gatekeeper residue at position 790 (T790M), bypass signaling via amplification of the gene encoding the RTK MET to activate a parallel pathway, and phenotypic alterations either by an epithelial–mesenchymal transition, or even by a transformation from NSCLC to small-cell lung cancer (Sequist *et al*, 2011). Clinical studies of acquired resistance provide clear rationales on how to develop improved strategies to prevent or overcome resistance, guiding the development of next-generation inhibitors addressing secondary target mutations, or informing new combinations aimed at co-targeting of bypass tracks. Preclinical modeling of acquired resistance to targeted therapies also yields key insights into target modifications or bypass mechanisms providing the capacity to substitute for the driver oncogene and serves as a powerful starting point when searching for clinical resistance mechanisms. As a word of caution, only a subset of the preclinical resistance mechanisms are actually found in the clinical setting. For example, initial attempts to predict resistance mechanisms to BRAF inhibitors focused on generating mutations in the BRAF gatekeeper residue that is analogous to the clinically relevant residues in EGFR (T790M), BCR-ABL (T315I), and ALK (L1196M). Indeed, it turned out that although engineering of BRAF T529 gatekeeper mutations does confer vemurafenib resistance *in vitro*, these mutations have never been reported in tumor samples from BRAF inhibitor-resistant patients (Whittaker *et al*, 2010). Thus, in this case, the design of second-generation compounds based on the preclinical findings would have been premature.

Massive cancer genome surveys supported by public or private funding are now rapidly expanding the catalogue of genetic aberrations linked to a broad range of cancer types. The cancer genome landscape in AML (The Cancer Genome Atlas Research Network, 2013) has informed new precision oncology approaches beyond the use of kinase inhibitors. Thus, in addition to TKIs targeting FLT3, small-molecule inhibitors are being developed to address AML subtypes harboring oncogenic variants of the gene encoding isocitrate dehydrogenase (IDH) 2 (e.g. AG-221; Stein *et al*, 2014) and the mixed-lineage leukemia gene MLL1 (Dot1L inhibitor EPZ-5676; Daigle *et al*, 2013; ClinicalTrials.gov identifiers: NCT02141828, NCT01684150). In addition to the genome-based discoveries of “actionable” cancer genes, several examples of cancer-specific vulnerabilities are poised to yield new therapeutic advances and deserve discussion in this context. In AML, the gene encoding the bromodomain-containing protein 4 (BRD4) was identified by an epigenetically focused systematic *in vivo* RNAi screen at the Cold Spring Harbor Laboratory as highly essential for promoting proliferation and blocking differentiation. BRD-containing proteins function by binding acetylated lysines on histone residues and recruiting protein complexes, thereby regulating gene expression by modulating heterochromatin. When using a BRD4 inhibitory “probe compound” (for definition, see Box 1), discovered at the Dana-Farber Cancer Institute, anti-leukemic effects across genomic AML subtypes were precisely recapitulated and found to largely depend on blocking the oncogenic transcription factor MYC (Zuber *et al*, 2011). Several companies have rapidly initiated discovery programs, and clinical trials probing the utility of BRD4 inhibitors for the treatment of AML are underway (Papavassiliou & Papavassiliou, 2014).

Prioritizing the best possible targets requires collaborative efforts at research institutions and hospitals to establish improved preclinical models.

A particularly good example of the contribution of modern molecular cell biology toward new treatment paradigms in AML is the field of cell cycle regulators. Here, the detailed understanding of how mitotic kinases such as polo-like kinase (Plk) 1 orchestrate mitosis, built over the years in basic academic research, inspired the industry to consider Plk1 inhibition as a potential therapeutic cancer target. Several small-molecule inhibitors have been developed that have enabled cell biologists to advance our understanding of Plk1 biology, and helped collaborating industry partners to develop Plk1 inhibition as an attractive therapeutic concept based on very efficient tumor cell killing and—in contrast to microtubule-targeting antimitotic agents—specificity for proliferating versus non-proliferating cells (Steegmaier *et al*, 2007; Taylor & Peters, 2008). Importantly, recent clinical data in AML patients demonstrated that the Plk1 inhibitor volasertib combined with chemotherapy was associated with higher response rates and improved event-free survival than chemotherapy alone (Döhner *et al*, 2014).

Finally, turning to gynecological tumors, a breakthrough discovery by two groups in the UK showed that inhibitors of the DNA repair enzyme poly(ADP) ribose polymerase I (PARP1) preferentially killed cancer cells harboring defects in the homologous recombination (HR) repair tumor suppressor proteins BRCA1 or BRCA2 (Bryant *et al*, 2005; Farmer *et al*, 2005). When HR is defective, alternative DNA repair mechanisms are utilized that then become dependent on PARP1. Key to the success of these studies have been the insights into DNA repair pathways made in basic academic research, as well as the availability of exquisite PARP inhibitor probe compounds pioneered in industry. The discovery also delivered strong support to the concept of “synthetic lethality”, based on which mutations that are harmless on their own, when compounded can kill a cancer cell. Subsequent studies established that olaparib, a potent PARP inhibitor, yielded notable clinical activity in *BRCA* mutation carriers with breast and ovarian cancers (Fong *et al*, 2009), providing the basis for ongoing trials (Moore *et al*, 2014).

## Challenges in advancing new precision cancer therapies

Recent surveys based on the sequencing of thousands of cancer genomes have yielded unprecedented insights into cancer genome landscapes (Vogelstein *et al*, 2013) and enabled the discovery of many new cancer genes, including several involved in cellular processes not previously thought to be causally linked to cancer (Garraway & Lander, 2013). In parallel, large-scale efforts, such as epigenomics, transcriptomics, proteomics, chemical genomics, and high-throughput functional screens have greatly increased our understanding of the underlying biology of cancer. While these transformative efforts continue to progress at a rapid pace, we perceive four key challenges going forward: how can we (i) prioritize the best possible targets; (ii) develop drugs against “undruggable” oncoproteins; (iii) restore tumor suppressor pathways; and (iv) identify highly effective drug combinations.

drug companies are now increasingly teaming up to clinically test targeted pathway inhibitor combinations.

Prioritizing the best possible targets requires collaborative efforts at research institutions and hospitals to establish improved preclinical models, including patient-derived xenografts, syngeneic models, and genetically engineered mouse models that better reflect the clinical situation (Toniatti *et al*, 2014). Furthermore, optimized validation tools enabling effective and versatile target knockdown, as well as reduced off-target effects (Fellmann *et al*, 2013), complemented by suitable chemical probes are most urgently needed to systematically assess potential cancer drivers. Importantly, strong cell biology and cellular signaling expertise will continue to prove crucial in exploring disease impact and in developing concepts for new precision medicines.

Overcoming technical hurdles to develop therapeutics against traditionally “undruggable” target classes remains a key issue. We see a trend that academic groups are taking up the challenge, supported by industry, to tackle some of the major culprits of cancer with innovative chemistry approaches. These include protein–protein interaction surfaces of highly validated targets, such as KRAS, MYC, p53, and beta-catenin, perhaps best illustrated by the recent report on KRAS(G12C) lead compounds (Ostrem *et al*, 2013). Academic/industry collaborations also feature prominently in current ambitions to broaden the scope of druggable cancer-driving targets, with a focus on newly emerging target classes involved in cancer epigenetics, metabolism, splicing, and protein homeostasis (Garraway & Lander, 2013). Based on precompetitive partnerships with multiple drug companies, the Structural Genomics Consortium is developing open-access chemical probes to target proteins that are involved in epigenetic signaling, while the Dundee Consortium currently focuses on components of the phosphorylation and ubiquitin systems (Mullard, 2011).

To fully exploit tumor suppressor pathways for genome-based therapies, we need to further intensify our efforts to find synthetic lethal drug targets akin to PARP. “Project Achilles” is an example of a collaborative effort between academia and industry partners to identify (tractable) molecular targets that address loss-of-function causing cancer mutations (Cheung *et al*, 2011). By using a large panel of well-characterized cancer cell lines or engineered isogenic cell lines that model the variability observed in patients in a defined genetic context, the goal is to systematically uncover genotype-dependent key cancer cell vulnerabilities.

Finally, a major challenge is the discovery and translation of highly effective drug combinations to further improve health outcomes in difficult-to-treat cancer types. Resistance (both primary and acquired) to current precision therapies is attributed to the genetic complexity and heterogeneity of tumors, clonal evolution, feedback loops in signaling pathways, and cellular plasticity which cumulatively result in a range of escape routes available to cancer cells. For example, the discovery that effective treatment of *BRAF*-mutated colorectal cancers requires inhibition of both BRAF and EGFR (Prahallad *et al*, 2012) has resulted in several combination trials (Bernards, 2014). Again, using a systematic approach, the combination of MEK and CDK4 inhibitors was found effective in preclinical *NRAS*-mutant melanoma models (Kwong *et al*, 2012), providing the basis for clinical combination trials (Johnson *et al*, 2014). Encouraged by such discoveries, drug companies are now increasingly teaming up to clinically test targeted pathway inhibitor combinations (Box 3).

**Box 3: Partnerships between companies to test drug combinations**

The combination of investigational cancer therapies into a single development program offers an attractive approach for generating more effective cancer treatments. This strategy allows the targeting of multiple cancer pathways, or addressing more than one key node in a single pathway to prevent or overcome intrinsic or acquired resistance (see Box 2). A single biomedical company rarely has adequate resources or the success rate to effectively targ*et all* major oncogenic pathways, their key nodes, and respective resistance mechanisms. To move forward efficiently, companies should collaborate rather than compete to be successful in developing highly effective combination therapies. Fortunately, we see a clear trend that such collaborative efforts are in fact happening. This started in 2009, with competitors AstraZeneca and Merck forming a partnership to evaluate a combination of AstraZeneca's MEK inhibitor and Merck's AKT inhibitor in multiple early-stage clinical trials. Since then, other top tier pharmaceutical companies have agreed on strategic collaborations to share drugs and development costs. Notable examples include partnerships between Merck KGaA and Sanofi on MEK and PI3K inhibitor combinations, as well as Roche and BMS on investigating Roche's vemurafenib in combination with BMS's ipilimumab, an immune-checkpoint inhibitor targeting cytotoxic T-lymphocyte activator-4 (CTLA-4), in patients with *BRAF*-mutated malignant melanoma. In the area of immune-checkpoint modulators, companies are teaming up for combinations centered around inhibitors of programmed cell death-1 (PD-1) combinations with other immunotherapies or with targeted therapies (Sheridan, 2014). We see it as very important for the entire drug development community to fully embrace and foster collaboration models to best advance investigational combination therapies for the benefit of cancer patients.

## A recipe for biomedical innovation

Collectively, innovation leading to effective new cancer therapies is driven by the convergence of basic scientific discoveries, rapidly improving genomic analysis technologies, and increasingly precise biomarker-guided development strategies. As illustrated, breakthrough innovation can equally well emerge from academia and industry and can arise both from “data first”, large-scale efforts and from creative, “hypothesis first”, modern molecular cell biology studies, often in a complementary and mutually beneficial manner.

Major advances can only be made if academic and company investigators team up for equal partnerships.

Biomedical scientists with a collaborative spirit, capable of networking across disciplines and beyond the classical academic and industry boundaries, and importantly those who are capable of integrating all relevant insights to address unmet medical needs, will be the true medical innovators. In our view, close academia/industry collaborations fuelled by open innovation are the most likely to succeed in generating and testing new therapeutic concepts and in translating the benefits to cancer patients. Thus, scientists and clinicians should be encouraged to engage in these interactions, despite some cultural barriers or the complications associated with managing intellectual property. To overcome these hurdles, truly innovative projects that—based on expertise and resources—would not be possible for either partner alone should be prioritized. Major advances can only be made if academic and company investigators team up for equal partnerships, take long-term views, share responsibilities and incentives, and build on their respective skill sets, with the ambition to succeed in a collective mission. Only with this integrated, dynamic, and open spirit can we jointly drive and accelerate the next stages of biomedical innovation to achieve meaningfully improved health outcomes for cancer patients.

## Conflict of interest

MAH has previously been compensated as a consultant and speaker for Roche and as a speaker for MSD and Amgen. NK is an employee of Boehringer Ingelheim.
